# Unparalleled Fecundity

**Published:** 1886-02

**Authors:** 


### UNPARALLELED FECUNDITY.

We extract the following from the recent letter of a Naples correspondent to the *Paris Register*:

The most extraordinary case of fecundity that I ever heard of came to my knowledge last week. About twenty-five miles from here, and by rail two or three stations beyond Pompeii, is the historical city of Nocera (the Nucera of the ancients). Twenty times have I passed through Nocera this past summer and autumn, and have always admired the wonderful productiveness of the fields, the vineyards and orchards. But here is something strictly true, which in human productiveness, rivals the exuberance of the soil, viz.: in the rione (or ward) of Liposta lives Maddalena Granata aged forty-seven who was married at the age of twenty-eight to a peasant —just nineteen years ago. Maddalena Granata has given birth to either dead or living, sixty-two children, fifty-nine of whom were males. She enjoys florid health, is robust and twenty-four hours after her last *accouchement* was ready to go out to her accustomed labor in the field. She has no hesitancy in conversing with any one about her extraordinary prolificness. Her physician, Dr. Raphael de Sanctis, of Nocera, says that there is not the least exaggeration in these statements. Has any one ever heard of such phenomenal fecundity in the whole history of maternity—sixty-two children, alive or dead, in nineteen years—*i. e.*, on an average twelve children every fourteen months! I leave it to your medical or surgical readers to make their researches and see if in all their statistics they can find a parallel case.

